# Predictive value of serum cystatin C for acute kidney injury in adults: a meta-analysis of prospective cohort trials

**DOI:** 10.1038/srep41012

**Published:** 2017-01-23

**Authors:** Zhenzhu Yong, Xiaohua Pei, Bei Zhu, Haichuan Yuan, Weihong Zhao

**Affiliations:** 1 Division of Nephrology, Department of Geriatrics, The First Affiliated Hospital of Nanjing Medical University, 300 Guangzhou Road, Nanjing 210029, Jiangsu, P.R. China

## Abstract

The role of serum cystatin C (Scys) for the detection of acute kidney injury (AKI) has not been fully discussed. This meta-analysis was aimed to investigate the overall diagnostic accuracy of Scys for AKI in adults, and further identify factors affecting its performance. Studies before Sept. 2016 were retrieved from PubMed, Embase, Web of Science and the Cochrane Library. A total of 30 prospective cohort studies (involving 4247 adults from 15 countries, 982 patients occurring AKI) were included. The revised Quality Assessment for Studies of Diagnostic Accuracy (QUADAS-2) tools demonstrated no significant bias had influenced the methodological quality of the included studies. Scys showed a high predictive power for all-cause AKI, that the area under the receiver operating characteristic curve was 0.89. The detailed assessment parameters, such as sensitivity, specificity, positive likelihood ratio, negative likelihood ratio and diagnostic odds ratio for Scys were 0.82, 0.82, 4.6, 0.22 and 21, respectively. Although Scys could be slightly influenced by the following factors: settings, AKI diagnostic criteria, ethnicity, determination method, age and gender, these factors above did not reach statistically significance. In conclusion, Scys could be a vital promising marker to screen out AKI.

Acute kidney injury (AKI) has been recognized as an independent risk factor for prolonged hospital stay, new-onset chronic kidney disease (CKD) and increased mortality rate^1^,^2^. Seriously, the prevalence of AKI is increasing in recent years^3^,^4^: nearly 3–20% for general inpatients, 30–60% for intensive care unit (ICU) patients.

To earlier and more accurate screen out the severe disease, diagnosis criteria for AKI have been updated three times: the RIFLE (Risk, Injury, Failure, Loss, End-Stage Kidney Disease) criteria in 2004 year, AKIN (Acute Kidney Injury Network) criteria in 2007 year, and the newly 2012 KDIGO (Kidney Disease Improving Global Outcomes) criteria^5^,^6^,^7^. It should be mentioned that all of the three criteria use the same kidney function assessment index: serum creatinine (Scr) and urine output.

However, both Scr and urine output are known as insensitive and nonspecific parameters for renal function evaluation. Thus, a great variety of bio-markers has been identified and then applied in the clinical settings in recent years^8^,^9^,^10^. Among the potential markers, serum cystatin C (Scys) performs a consistent accuracy in various conditions. For both the healthy individuals and CKD patients, Scys has ever been proposed as a superior marker to Scr to evaluate glomerular filtration rate (GFR)^11^,^12^.

Recently, based on its physiological metabolism characteristics, that the life cycle of Scys is merely half of that of Scr (1.5–2 hours vs. 4 hours). Namely, once renal function is fluctuating, Scys changes much earlier than Scr^13^,^14^. Zhang, *et al*.^15^ performed a meta-analysis to compare serum and/or urine cystatin C for diagnosis of AKI, and then they found Scys appeared to be a better biomarker in the prediction of AKI.

Since then, concerns focused on Scys for AKI prediction have been accelerating. However, with accumulating evidence, conflicting results have raised. Wan *et al*.^16^ reported that the predict value (the area under the receiver operating characteristic curve, AUROC) of Scys was 0.974, with high sensitivity and specificity, which were similar in Liu’s^17^, Yim’s^18^ and other studies. In contrast, another studies indicated a negative results. GaygIsIz *et al*.^19^ and Martensson *et al*.^20^ found that the predict values (AUROC) of Scys was 0.67, with low sensitivity and specificity. Based on these controversial results, we conducted the present meta-analysis to investigate the overall diagnostic accuracy of Scys for AKI, and further identify which factors affecting its performance.

## Results

### Literature search

**Figure 1:**
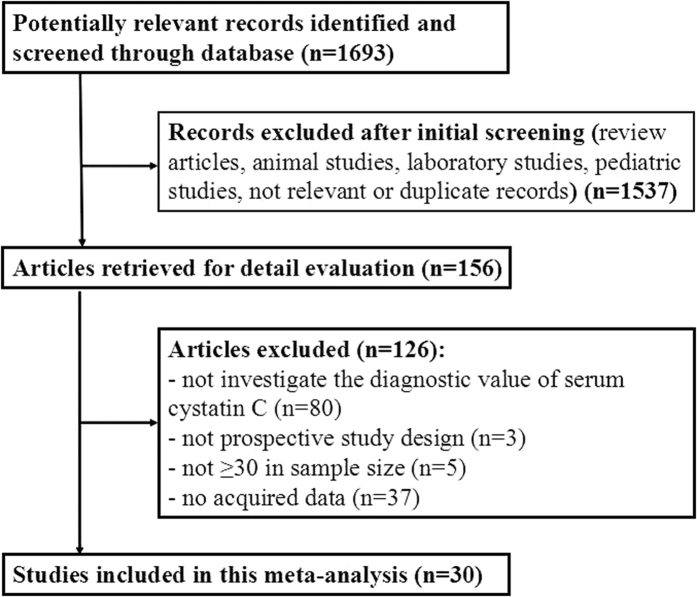
Flow chart of study selection.

Our research initially identified 1693 citations, among which, 1537 were excluded as they were review articles, animal studies, laboratory reports, pediatric studies, not relevant and duplicate records. Thirty studies^16^,^17^,^18^,^19^,^20^,^21^,^22^,^23^,^24^,^25^,^26^,^27^,^28^,^29^,^30^,^31^,^32^,^33^,^34^,^35^,^36^,^37^,^38^,^39^,^40^,^41^,^42^,^43^,^44^,^45^ finally met the inclusion criteria via full-text evaluation from 156 potentially eligible citations. Seven studies^21^,^22^,^23^,^24^,^25^,^26^,^27^ were selected from the previous meta-analysis (Zhang, *et al*. 2011)^15^ and an additional 23 studies^16^,^17^,^18^,^19^,^20^,^28^,^29^,^30^,^31^,^32^,^33^,^34^,^35^,^36^,^37^,^38^,^39^,^40^,^41^,^42^,^43^,^44^,^45^ were complemented in the present meta-analysis. A flow chart of the identification and selection process is shown in Fig. 1.

### Subjects characteristics and quality assessment

The main characteristics of the included studies were summarized in Tables 1 and 2. A total of 4,247 patients (mean age 61.6 years, male 70.9%) from 15 countries were enrolled in the 30 studies. The overall AKI incidence was 23.1% (982/4247, varied from 6.0% to 54.3%). The top three settings prone to AKI were 32.3% after cardiac surgery, 28.5% in ICU/cardiac care unit (CCU) and 13.8% in contrast-induced nephropathy (CIN). The elderly and non-elderly patients suffered from an almost similar AKI prevalence (23.1% vs. 22.6%, *P* > 0.05).

**Figure 2:**
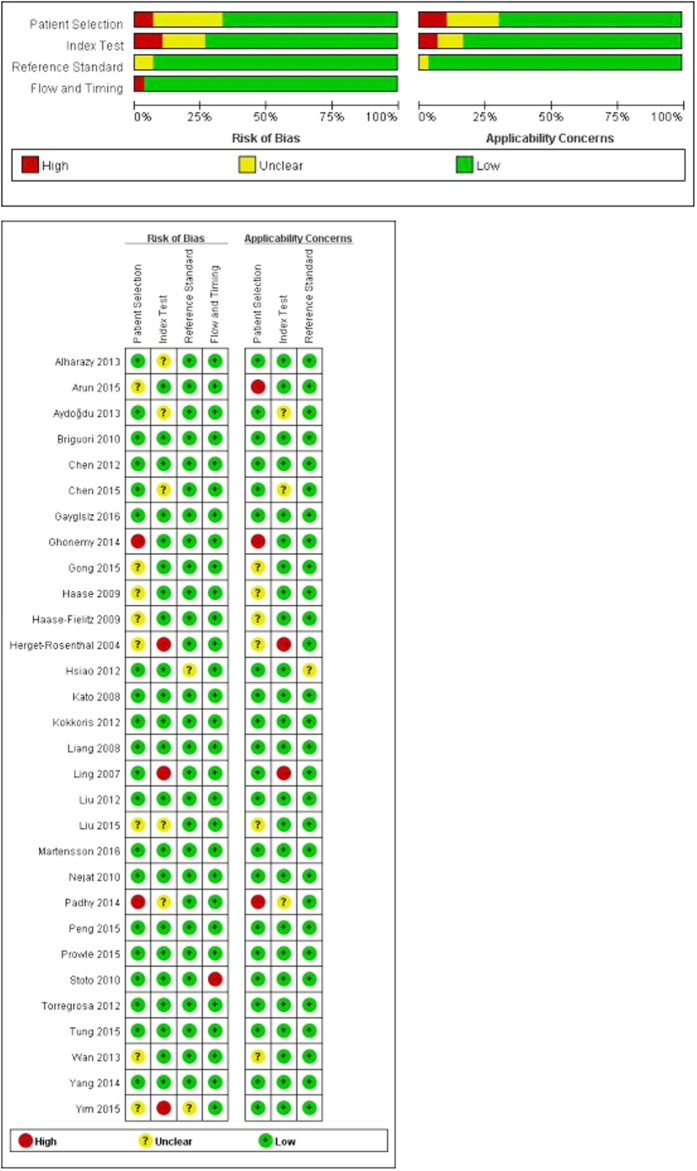
Assessment of the methodological quality of the selected studies by the Quality Assessment of Diagnostic Accuracy Studies tool, version 2 (QUADAS-2).

The second version of the Quality Assessment of Diagnostic Accuracy Studies (QUADAS-2) plot demonstrated no significant bias had influenced the methodological quality of the included studies (Fig. 2).

### Predictive value of Scys for AKI

**Figure 3:**
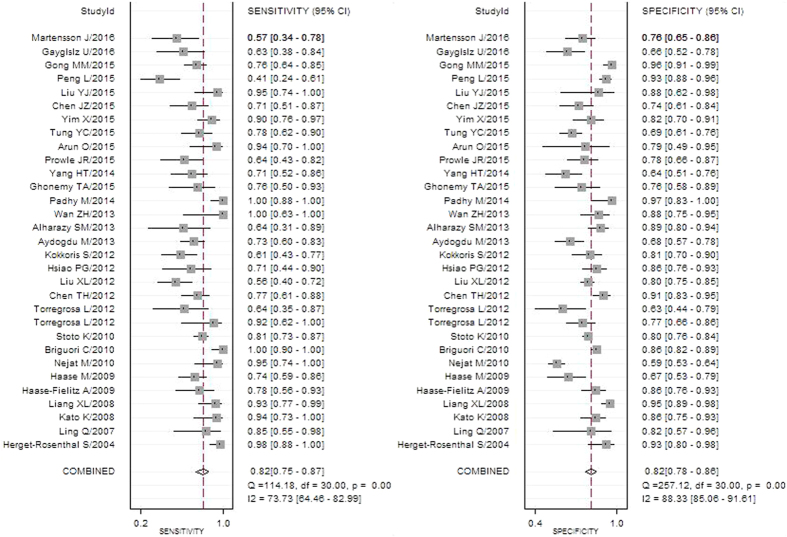
Forest plot of the pooled sensitivity and specificity of serum cystatin C to predict all-cause acute kidney injury.

**Figure 4:**
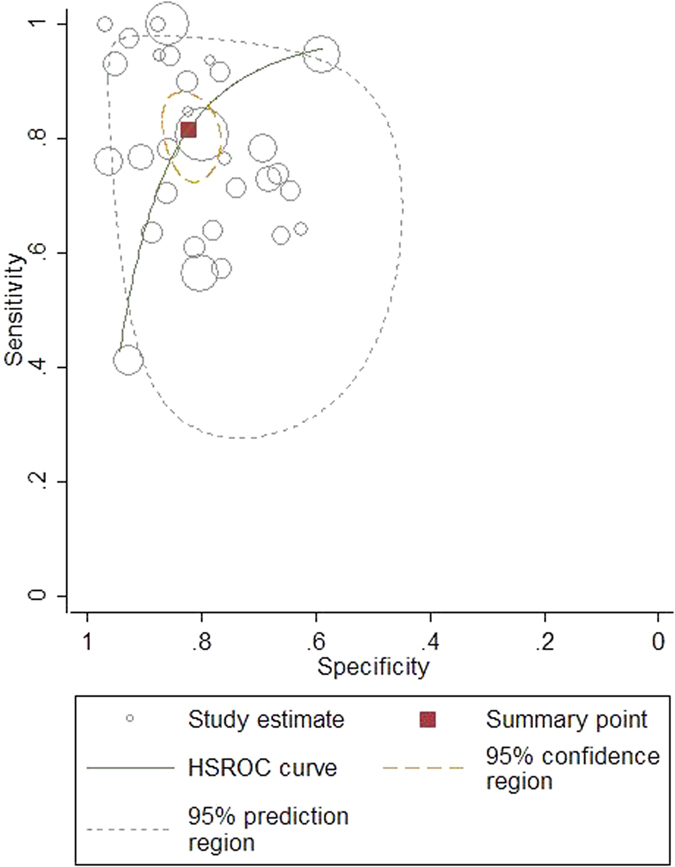
Hierarchical summary receiver operating characteristic (HSROC) plot of serum cystatin C to predict acute kidney injury across all settings.

The pooled diagnostic accuracy of Scys was listed in Table 3 and Fig. 3. The overall diagnostic sensitivity and specificity was 0.82 (95% CI: 0.75 to 0.87) and 0.82 (95% CI: 0.78 to 0.86), respectively. The pooled positive and negative likelihood ratios were 4.6 (95% CI: 3.6–5.9) and 0.22 (95% CI: 0.16–0.31), respectively and the DOR was 21 (95% CI: 12–35). The overall area under the receiver operating characteristic curve (AUROC) reached 0.89. All the results above revealed a good diagnostic accuracy of Scys to screen out AKI (Fig. 4).

### Threshold analysis and meta-regression analysis

The Spearman correlation coefficient between the pooled sensitivity and 1-specitity was −0.277 (P = 0.131), indicating no threshold effect. Meta-regression analysis showed the following factors irrelevant with accuracy of Scys for AKI: settings, diagnostic criteria, region, Scys determination method, participant mean age, gender, sample size (Table 3).

### Influence factors affecting Scys of AKI

Various Scys blood sampling point-in-time, cut-off value, and determination method resulted in various Scys predictive value for AKI by subgroup analysis (Tables 1,2,3,4). Foremost, Twenty-four hours after AKI occurrence to adopt the blood seems to be an optimal time, with sensitivity of 0.82, specificity of 0.83, DOR of 23, and AUROC of 0.89 (Table 4). Besides, 50% elevated from baseline could be an ideal cut-off value to predict AKI, with AUROC 0.99 (Table 2). Last but not the least, PETIA performed better than other two determination methods, with sensitivity 0.76, specificity 0.87 and AUROC 0.90 (Table 3). In addition, several factors other than Scys assay itself were also analyzed in this study, such as AKI diagnostic criteria, region, gender, age and sample size and *et al*. Based on the newly KDIGO criteria, Scys performed the best accuracy, with sensitivity 0.78, specificity 0.90 and AUROC 0.92 (Table 3). However, these factors mentioned above were not the origin of possible sources of heterogeneity by meta-regression analysis.

### Publication bias

**Figure 5:**
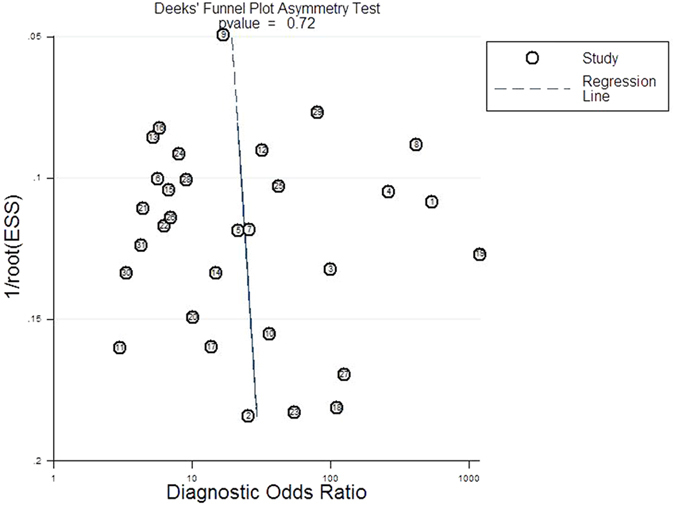
Deeks’ funnel plot to analyze the likelihood of publication bias.

No publication bias and high symmetry of the included studies were proved by Deeks’ funnel plot asymmetry test (*P* = 0.72; Fig. 5).

## Discussion

The overall AKI incidence in this study was 23.1% (982/4247), similar to the prevalence reported in Siew’ study4, indicating the disease is still not in control and prevented. The mean age in this meta-analysis achieved 61 years old, demonstrating more attention should be taken on the susceptible population: the elderly. Various setting, various AKI incidence. The top three settings prone to AKI were cardiovascular surgery, ICU/CCU and radiology intervention department.

Facing to the severe reality, early diagnosis is crucial to prevent and relieve the prognosis of AKI. Scys has been known to be an ideal marker to assess renal function in CKD patients^46^,^47^. Whether it is a satisfactory marker to predict AKI is still in debate. Thus, to comprehensively and objectively evaluate the value of Scys predicting AKI, this meta-analysis set a rigorous inclusion and exclusion criteria at the very start. One of the essential selected condition should be prospective cohort studies. After literature searching, 30 studies finally were included. The pooled sensitivity, specificity and AUROC of Scys was 0.82, 0.82 and 0.89, respectively. These diagnostic efficiency demonstrated that Scys would be an excellent bio-marker for the all-cause AKI prediction.

Further subgroups analysis indicated several influence factors should be noted. Different Scys blood sampling point-in-time, cut-off value, and determination method, different Scys predictive value for AKI. If an AKI event would occur, it could be suggested that Scys should be determined by PETIA method at 24-hours after the possible AKI event, referring the diagnostic criteria-50% elevated from baseline. Compared with the previous studies results, this advice is rational and acceptable. The blood sampling point-in-time was another focus. Among the various time point, 24-hours point after AKI might be a preferable selection.

Otherwise, factors potentially influencing Scys were also assessed in this study. Three main criteria to diagnose AKI were presented in Table 3. The newly KDIGO criteria performed an increased diagnostic accuracy in Thomas’ study^48^. The subgroup analysis in this study also confirmed its superiority, that the newly KDIGO criteria showed higher specificity and AUROC than the RIFLE criteria and AKIN criteria.

As reported, the most common cause of AKI is acute tubular necrosis (ATN), which could be caused by prolonged hypotension, sepsis, surgery, nephrotoxic medications, and contrast media in hospitalized patients^49^,^50^. Among the three main causes of AKI in this meta-analysis, Scys performed the best accuracy in CIN-AKI. The probable reason might be that kidney injury and hemodynamic disorder induced by CIN-AKI is less serious and complicate than that by CS-AKI and ICU-AKI. CIN could be the most simple, but also the most important AKI model to ascertain the value of Scys. To our knowledge, CIN is the third leading cause of AKI in hospitalized patients^51^,^52^. There is a variety of novel bio-markers have been proposed to diagnose CIN. Among them, Neutrophil gelatinaseassociated lipocalin (NGAL), interleukin-18 (IL-18) and Scys are the most known promising bio-markers^53^,^54^. However, the former two biomarker determination method have not yet been established in clinical laboratories. Thus, according to the results of this study, Scys could be the optimal marker predicting various AKI.

It should be mentioned in the end, the same as the previous CKD studies proved^55^, Scys was not significantly influenced by gender and age in this AKI-related study, as well. Moreover, although settings, AKI diagnostic criteria, race and assay method might play a little bit of influence on the accuracy of Scys, it did not reach statistical significance. Thus, these results above showed that Scys could be a nice marker, not only for CKD diagnosis, but for AKI prediction.

For all meta-analyses, heterogeneity is a potential problem when interpreting the results. The *I*^*2*^ statistic was 96% in our meta-analysis, indicating significant heterogeneity across the included studies. One major source of heterogeneity is the threshold effect in which different cut-offs are used in the included studies. The Spearman correlation analysis in our study indicated no threshold effect related heterogeneity exit. Furthermore, meta-regression analysis results revealed that factors potentially affecting Scys did not participate in the heterogeneity (p > 0.05; Table 3). Thus, we considered that the heterogeneity may be related to additional factors, such as specified ethnicity (except from the two race in this study), kidney function, and etc. However, these factors is difficult to unify and analyze.

In summary, this meta-analysis demonstrated that Scys shows a good diagnostic performance for predicting all-cause AKI. Several factors could affect the predictive value of Scys for AKI, but not reach significant differences. More randomized controlled trials in multicenter are in need to further investigate the accuracy of Scys.

## Methods

### Data sources and search strategy

In accordance with the Preferred Reporting Items for Systematic reviews and Meta-Analyses (PRISMA) guidelines^56^, we searched PubMed, EMBASE, Web of Science and the Cochrane Library from the inception to September 2016.

The following terms were used: “AKI, acute kidney injury, acute renal failure, acute renal insufficiency, acute renal dysfunction and cystatin C”. References of the selected studies were further screened manually to identify whether additional eligible articles were available or not.

### Study selection

The inclusion criteria of this study were composed of the following characteristics: (1) prospective cohort study, (2) adults, (3) sample size ≥ 30, (4) original data of sensitivity and specificity, (5) AKI diagnostic criteria. If any disagreement existed, two investigators would check and discuss about the full text.

Authors were contacted when there were incomplete or missing data. Ethics approval and patients consent were not in need for this study.

### Data extraction and quality assessment

Two investigators (Z.Z.Y. and X.H.P.) independently extracted information from each article using a standardized collection form. Collected parameters included the first author, publication year, clinical setting, region, age, gender, AKI diagnostic criteria, Scys determination method, Scys cut-off value, sensitivity and specificity. Differences were resolved by consensus or the third researcher (W.H.Z.).

We investigated the methodological quality of the present study using the second version of the Quality Assessment of Diagnostic Accuracy Studies (QUADAS-2)^57^. QUADAS-2 assesses the risk of bias and applicability in four domains: Patient selection (consecutive or random sample enrolled, case–control design and inappropriate exclusions avoided); Index test (blinded interpretation of the Rules); Reference standard (correctly excluded a fracture and blinded interpretation); and Flow and timing (appropriate interval between application of the Rules and reference standard, all patients received the reference standard and were included in the analysis).

### Statistical analysis

A bivariate meta-analytic approach was used to pool sensitivity, specificity, DOR, PLR, and NLR. Subsequently, the respective hierarchical summary receiver operating characteristic (HSROC) curves was constructed to plot sensitivity versus specificity, and then calculate the area under the curve. The highest Youden index (sensitivity + 1-specificity) of every included studies was chosen to end pooled in various Scys measurement times^58^. We used the *I*^*2*^ statistic to evaluate the heterogeneity^59^, and the *I*^*2*^ > 75% is supposed of significant heterogeneity, the threshold analysis and meta-regression analysis were further used to identify possible sources of heterogeneity. Publication bias was estimated by Deeks’ funnel plot asymmetry test^60^. All the data processing and analysis were performed using the midas and metandi commands of Stata/SE version 12.0 (Stata Corp LP, College Station, TX) and Meta-Disc 1.4 for Windows (XI Cochrane Colloquium, Barcelona, Spain). QUADAS-2 quality assessment was descriptively analyzed using Review Manager 5.3 (The Cochrane Collaboration, Copenhagen, Denmark). P < 0.05 was considered of statistical significance.

## Additional Information

**How to cite this article**: Yong, Z. *et al*. Predictive value of serum cystatin C for acute kidney injury in adults: a meta-analysis of prospective cohort trials. *Sci. Rep.* **7**, 41012; doi: 10.1038/srep41012 (2017).

**Publisher's note:** Springer Nature remains neutral with regard to jurisdictional claims in published maps and institutional affiliations.

## Figures and Tables

**Table 1:** Basic characteristics of the selected AKI studies for Scys.

Study	Country	Clinical setting	No. of patients	AKI incidence (%)	Mean age (y)	Males (%)	Definition of AKI	Scys assay method
Herget-Rosenthal S.^21^	Germany	ICU	85	51.8	66.6	63.9	RIFLE-R	Immunonephelometric assay
Ling Q.^22^	China	LTx	30	43.3	47	90	GFR < 80 mL/min/1.73 m^2^	NR
Kato K.^28^	Japan	CIN	87	20.7	67	71.3	Scr↑ > 25% or > 0.5 mg/dL within 48 h	PENIA
Liang X. L.^23^	China	CS	132	22.0	NR	NR	RIFLE ≥ R	PETIA
Haase-Fielitz. A.^29^	Australia	CS	100	23.0	69.5	61	RIFLE ≥ R	Immunonephelometric assay
Haase M.^24^	Australia	CS	100	46.0	71	61	AKIN ≥ 1	Immunonephelometric assay
Nejat M.^25^	New Zealand	ICU	318	6.0	60	61.1	RIFLE ≥ R	PENIA
Briguori C.^26^	Italy	CIN	410	8.3	70	83.9	Scr↑ ≥ 0.3 mg/dL from baseline	Dade Behring N Latex Scys assay
Stoto K.^27^	Portugal	ED	616	21.1	59.1	62.7	RIFLE ≥ R & AKIN ≥ 1	PENIA
Torregrosa L.^30^	Spain	CIN	89	13.5	62.6	75.3	RIFLE ≥ R	Immunonephelometric assay
	Spain	CS	46	30.4	68.8	73.9	RIFLE ≥ R	Immunonephelometric assay
Chen T. H.^31^	Taiwan	CCU	150	28.7	66	75.3	AKIN ≥ 1	ELISA
Liu X. L.^32^	China	CIN	311	12.5	58.9	63.7	KDIGO	ELISA
Hsiao P. G.^33^	Taiwan	AMI	96	17.7	63	90.6	AKIN ≥ 1	ELISA
Kokkoris S.^34^	Greece	ICU	100	36.0	49* & 63*	57	RIFLE ≥ R	Immunonephelometric assay
Aydoğdu M.^35^	Turkey	ICU	151	41.2	68.1	64.9	RIFLE ≥ R	PENIA
Alharazy S. M.^36^	Malaysia	CIN	100	11.0	60.4	79	Scr↑ ≥ 25% from baseline in 48 hours	PENIA
Wan Z. H.^16^	China	ACLF	56	14.3	44	71.4	AKIN ≥ 1	PENIA
Padhy M.^37^	India	CIN	60	50.0	55.9	73.3	KDIGO	ELISA
Ghonemy T. A.^38^	Egypt	CS	50	34.0	44.4	64	RIFLE ≥ R	ELISA
Yang H. T.^39^	Korea	MBI	90	34.4	49.3	85.6	RIFLE ≥ R	Immunoturbidimetric assay
Prowle J. R.^40^	Australia	CPB	93	26.9	70*	69	RIFLE ≥ R	Immunonephelometric assay
Arun O.^41^	Turkey	CS	30	53.3	71.9	73.3	KDIGO	Immunonephelometric assay
Tung Y. C.^42^	Taiwan	ED	189	19.6	62.3	86.6	AKIN ≥ 1	ELISA
Yim H.^18^	Korea	BICU	97	41.2	47	80.4	AKIN ≥ 1	NR
Chen J. Z.^43^	China	PNE	89	31.5	48.9	66.3	AKIN ≥ 1	ELISA
Liu Y. J.^17^	China	CS	35	54.3	52.2	34.3	AKIN ≥ 1	PETIA
Peng L.^44^	China	CIN	196	14.8	70.4	68.4	KDIGO	PETIA
Gong M. M.^45^	China	ICU	176	40.3	55.1	61.9	KDIGO	ELISA
GaygIsIz U.^19^	Turkey	ICU	72	26.4	64.6	72.2	RIFLE ≥ R	PENIA
Martensson J.^20^	Australia	ICU	93	22.6	50* & 66*	71	KDIGO	PETIA

Abbreviations: ACLE, acute-on-chronic liver failure; AMI, acute myocardial infarction; AKI, acute kidney injury; BICU, burn intensive care unit; CCU, coronary care unit; CIN, contrast-induced nephropathy; CPB, cardiopulmonary bypass; CS, cardiac surgery; ED, emergency department; ELISA, enzyme-linked immunosorbent assay; GFR, glomerular filtration rate; KDIGO, Kidney Disease: Improving Global Outcomes; LTx, liver transplantation; MBI, major burn injury; NR, not reported; PENIA, particle-enhanced nephelometric immunoassay; PETIA, particle-enhanced turbidimetric immunoassay; PNE, Partial nephrectomy; RIFLE, risk-injury-failure-loss-end stage renal disease; Scr, serum creatinine; Scys, serum cystatin C.

*Median age (year).

**Table 2:** The accuracy of Scys at various blood sampling point-in-time and cut-off value.

Study	Blood sampling point-in-time	Scys cutoff value	Test results				Sensitivity (%)	Specificity (%)	AUROC (95% CI)
			TP	FP	FN	TN			
Herget-Rosenthal S.^21^	On day after kidney injury	↑ ≥ 50% from baseline	43	3	1	38	98	93	0.99(0.98, 1.00)
	24 h before kidney injury	↑ ≥ 50% from baseline	36	2	8	39	82	95	0.97(0.94, 0.99)
	24 h before kidney injury	↑ ≥ 50% from baseline	24	2	20	39	55	95	0.82(0.71, 0.92)
Ling Q.^22^	Post-Tx d 1,4, &7	1.57 mg/L	11	3	2	14	84.6	84.5	0.94(0.86, 0.98)
Kato K.^28^	Before,1,2,3 days after catheterization	1.2 mg/L	17	10	1	59	94.7	84.8	0.93
Liang X. L.^23^	Postoperative d1	↑ ≥ 50% from baseline	27	5	2	98	92	95	0.99(0.98, 1.01)
Haase-Fielitz A.^29^	On ICU admission	1.1 mg/L	18	11	5	66	77	86	0.83(0.68, 0.98)
	24 h after CPB	1.2 mg/L	21	28	2	49	91	64	0.84(0.75, 0.93)
Haase M.^24^	6 h after CPB	1.1 mg/L	34	18	12	36	74	67	0.76(0.61, 0.91)
Nejat M.^25^	On ICU admission	0.8 mg/L	18	123	1	176	95	59	0.80(0.71, 0.88)
Briguori C.^26^	24 h after CM exposure	↑ ≥ 10% from baseline	34	53	0	323	100	85.9	0.92
Soto K^27^	On ED admission	0.98 mg/L	106	113	24	373	81.4	76.7	0.87(0.83, 0.90)
	6 h after ED admission	0.98 mg/L	106	112	24	374	81.6	77.0	0.87(0.83, 0.91)
	12 h after ED admission	0.98 mg/L	106	104	24	382	81.6	78.5	0.88(0.84, 0.91)
	24 h after ED admission	0.98 mg/L	103	109	27	377	79.5	77.5	0.86(0.82, 0.90)
	48 h after ED admission	0.98 mg/L	105	97	25	389	81	80.1	0.87(0.83, 0.91)
Torregrosa L.^30^	12 h after CAG	0.8 mg/L	11	18	1	59	89	76	0.87(0.68, 1.06)
	12 h after CS	0.8 mg/L	9	12	5	20	64	64	0.68(0.46, 0.88)
Chen T. H.^31^	On CCU admission	1.8 mg/L	33	10	10	97	77	91	0.90(0.83, 0.96)
Liu X. L.^32^	On hospital admission	475 ng/mL	22	54	17	218	57.1	80.1	0.63(0.53, 0.73)
	24 h after CM administration	503 ng/mL	22	108	17	164	57.1	60.2	0.63(0.54, 0.72)
Hsiao P. G.^33^	On 24 h of AMI after PCI	1364 mg/L	12	11	5	68	69.2	85.9	0.864
Kokkoris S.^34^	On ICU admission	1.04 mg/L	22	12	14	52	61.1	81.2	0.75(0.65, 0.83)
Aydoğdu M.^35^	Within first 24 h of ICU admission	1.5 mg/L	46	28	17	60	73	68	0.82
Alharazy S. M.^36^	At 24 h after CM exposure	0.19 mg/L	7	10	4	79	63.64	88.76	0.80(0.70, 0.87)
Wan Z. H.^16^	On Center admission	1.21 mg/L	8	6	0	42	100	87.5	0.97(0.85, 1.00)
Padhy M.^37^	0 h of angioplasty procedure	0.504 mg/L	20	11	10	19	66	63	0.70
	4 h of angioplasty procedure	0.517 mg/L	20	10	10	20	66.7	66.6	0.72
	24 h of angioplasty procedure	0.994 mg/L	30	1	0	29	100	96.7	1.00
	48 h of angioplasty procedure	0.961 mg/L	28	1	2	29	93.3	96.7	0.99
Ghonemy T. A.^38^	3 h after CS	2.65 ng/dL	9	9	8	24	54.7	72.7	NR
	6 h after CS	2.65 ng/dL	13	8	4	25	75.2	75.8	NR
Yang H. T.^39^	12 h from admission	0.70 mg/L	22	21	9	38	70.4	65.2	0.75
	24 h from admission	0.75 mg/L	16	23	15	36	50.0	61.8	0.73
Prowle J. R.^40^	4.5 h after CPB	1.24 mg/L	19	25	6	43	76.0	63.2	0.69(0.56, 0.82)
	24 h after CPB	1.57 mg/L	16	15	9	53	64.0	77.9	0.72(0.59, 0.85)
Arun O.^41^	At 1 h after CS	0.76 mg/dL	12	5	4	9	75	65	0.74
	At 12 h after CS	0.98 mg/dL	12	3	4	11	75	80	0.77
	At 24 h after CS	0.83 mg/dL	15	3	1	11	93	79	0.79
	At 48 h after CS	0.73 mg/dL	13	4	3	10	81	72	0.72
Tung Y. C.^42^	On ED admission	1.6 mg/L	29	47	8	105	79	69	0.73(0.63, 0.81)
Yim H.^18^	At 7 d after Burn	0.75 mg/L	31	14	9	43	76.3	75.4	0.81(0.71, 0.91)
	At 14 d after Burn	0.85 mg/L	36	10	4	47	89.5	82.5	0.91(0.84, 0.97)
Chen J. Z.^43^	At 24 h after PNE	0.98 mg/L	20	16	8	45	71.4	73.8	0.79(0.70, 0.89)
	At 48 h after PNE	1.005 mg/L	19	21	9	40	67.9	65.6	0.76(0.66, 0.86)
Liu Y. J.^17^	0 h after CS	0.965 mg/L	14	4	5	12	73.7	75.0	0.74(0.57, 0.91)
	4 h after CS	1.150 mg/L	15	4	4	12	78.9	75.0	0.86(0.74, 0.98)
	24 h after CS	1.275 mg/L	18	2	1	14	94.7	87.5	0.97(0.97, 1.02)
	48 h after CS	1.405 mg/L	18	2	1	14	94.7	87.5	0.97(0.93, 1.02)
	72 h after CS	1.380 mg/L	18	3	1	13	94.7	81.2	0.94(0.87, 1.02)
Peng L.^44^	24 h after PCI	↑ ≥ 15% from baseline	10	10	19	157	34.48	94.01	0.66(0.55, 0.77)
	48 h after PCI	↑ ≥ 15% from baseline	12	12	17	155	41.38	92.86	0.78(0.70, 0.87)
Gong M. M.^45^	On ICU admission	1.54 mg/L	54	4	17	101	76.1	96.2	0.90(0.86, 0.95)
GaygIsIz U^19^	within the first 24–48 h of ICU admission	0.94 mg/L	12	18	7	35	63	66	0.67 (0.53, 0.81)
Martensson J.^20^	within 48 h of ICU admission	1.1 mg/l	12	17	9	55	55	76	0.67 (0.54-0.81)

Abbreviations: AKI, acute kidney injury; AUROC, the area under the receiver operating characteristic curve; CAG, coronary angiography; CM, contrast medium; CPB, cardiopulmonary bypass; CS, cardiac surgery; DOR, diagnostic odds ratio; Scys, serum cystatin C; TP, true positive; FP, false positive; FN, false negative; TN, true negative; Tx, transplant; ICU, intensive care unit; ED, emergency department; PCI, percutaneous coronary intervention; PNE, Partial nephrectomy; NR, not reported.

**Table 3:** Pooled diagnostic accuracy of Scys in various AKI subgroup studies.

Settings	AKI Pts/Total Pts; No. of studies	Sensitivity (95% CI)	Specificity (95% CI)	DOR (95% CI)	AUROC (95% CI)	*I*^*2*^	Likelihood Ratio (95% CI)		RDOR	P Value
							Positive	Negative		
Across all settings	982/4247; 30	0.82 (0.75, 0.87)	0.82 (0.78, 0.86)	21 (12, 35)	0.89 (0.86, 0.91)	96	4.6 (3.6, 5.9)	0.22 (0.16, 0.31)		
Subgroups										
1.AKI setting:										
CS-AKI	189/586; 8	0.81 (0.71, 0.89)	0.82 (0.72, 0.89)	20 (7, 57)	0.89 (0.86, 0.91)	0	4.5 (2.7, 7.8)	0.23 (0.13, 0.39)	1.49 (0.65, 3.40)	0.327
ICU/CCU-AKI	340/1194; 9	0.77 (0.66, 0.85)	0.82 (0.72, 0.89)	16 (6, 38)	0.86 (0.83, 0.89)	89	4.3 (2.5, 7.4)	0.28 (0.18, 0.44)		
CIN	173/1253; 7	0.90 (0.61, 0.98)	0.87 (0.82, 0.90)	61(10, 388)	0.90 (0.88, 0.93)	95	6.7 (4.7, 9.7)	0.11 (0.02, 0.57)		
CIN	173/1253; 7	0.90 (0.61, 0.98)	0.87 (0.82, 0.90)	61(10, 388)	0.90 (0.88, 0.93)	95	6.7 (4.7, 9.7)	0.11 (0.02, 0.57)	2.24 (0.65, 7.79)	0.194
Exp-CIN	809/2994; 23	0.79 (0.74, 0.84)	0.81 (0.75, 0.85)	16(10, 26)	0.87 (0.84, 0.89)	85	4.1 (3.1, 5.4)	0.26 (0.20, 0.34)		
2.AKI diagnostic criteria:										
KDIGO	206/866; 6	0.78 (0.49, 0.93)	0.90 (0.81, 0.95)	31 (6, 174)	0.92 (0.90, 0.94)	77	7.7 (3.4, 17.7)	0.25 (0.09, 0.70)	0.60 (0.29, 1.22)	0.147
AKIN ≥ 1	238/812; 8	0.81 (0.74, 0.86)	0.81 (0.74, 0.87)	18 (9, 35)	0.86 (0.83, 0.89)	0	4.3 (3.0, 6.4)	0.24 (0.17, 0.34)		
RIFLE ≥ R	284/1241; 11	0.75 (0.67, 0.82)	0.76 (0.67, 0.82)	10 (5, 18)	0.82 (0.72, 0.85)	64	2.1 (2.2, 4.4)	0.32 (0.23, 0.46)		
3.region:										
Asia	578/2197; 20	0.81 (0.73, 0.87)	0.85 (0.80, 0.89)	24 (12, 45)	0.90 (0.87, 0.92)	93	5.3 (3.8, 7.5)	0.23 (0.15, 0.33)	1.69 (0.60, 4.78)	0.307
Non-Asia	404/2050; 10	0.83 (0.70, 0.91)	0.78 (0.71, 0.83)	17 (7, 43)	0.85 (0.82, 0.88)	82	3.7 (2.7, 5.2)	0.22 (0.11, 0.42)		
4.Scys assay:										
PETIA	129/546; 5	0.76 (0.51, 0.91)	0.87 (0.74, 0.94)	21 (4, 103)	0.90 (0.87, 0.92)	88	5.8 (2.5, 13.5)	0.27 (0.11, 0.67)	1.21 (0.56, 2.60)	0.614
PENIA	484/2043; 14	0.82 (0.31, 0.81)	0.78 (0.72, 0.83)	16 (8, 31)	0.86 (0.83, 0.89)	62	3.8 (2.8, 5.0)	0.24 (0.15, 0.37)		
ELISA	282/1121; 8	0.77 (0.66, 0.85)	0.86 (0.77, 0.92)	21 (8, 55)	0.88 (0.85, 0.90)	72	5.6 (3.0, 10.3)	0.27 (0.17, 0.42)		
5.participant mean age:										
≤60 years	445/1928; 12	0.85 (0.75, 0.91)	0.82 (0.74, 0.88)	26 (11, 61)	0.90 (0.87, 0.93)	89	4.8 (3.1, 7.4)	0.19 (0.11, 0.33)	1.24 (0.43, 1.56)	0.684
>60 years	451/1994; 15	0.80 (0.70, 0.88)	0.82 (0.76, 0.88)	18 (9, 36)	0.88 (0.84, 0.90)	93	4.4 (3.2, 5.9)	0.24 (0.15, 0.38)		
6.male rate:										
≤70%	604/2512; 14	0.78 (0.67, 0.86)	0.83 (0.78, 0.88)	18 (9, 36)	0.88 (0.85, 0.91)	94	4.7 (3.4, 6.5)	0.26 (0.17, 0.41)	0.96 (0.37, 2.49)	0.925
>70%	349/1603; 15	0.83 (0.75, 0.90)	0.80 (0.73, 0.85)	20 (10, 41)	0.88 (0.85, 0.91)	77	4.1 (3.0, 5.7)	0.21 (0.13, 0.33)		
7.sample size:										
≤100	488/1598; 20	0.82 (0.74, 0.88)	0.81 (0.77, 0.85)	20 (10, 39)	0.88 (0.85, 0.91)	42	4.4 (3.3, 5.8)	0.22 (0.14, 0.33)	0.59 (0.21, 1.65)	0.304
>100	494/2649; 10	0.81 (0.67, 0.89)	0.85 (0.76, 0.91)	23 (10, 54)	0.90 (0.87, 0.92)	96	5.3 (3.3, 8.6)	0.23 (0.13, 0.40)		

Abbreviations: AKI, acute kidney injury; AUROC, the area under the receiver operating characteristic curve; CCU, coronary care unit; CIN, contrast-induced nephropathy; CS, cardiac surgery; DOR, diagnostic odd ratio; ELISA, enzyme-linked immunosorbent assay; KDIGO, Kidney Disease: Improving Global Outcomes; PENIA, particle-enhanced nephelometric immunoassay; PETIA, particle-enhanced turbidimetric immunoassay; Pts, patients; RIFLE, risk-injury-failure-loss-end stage renal disease; Scr, serum creatinine; Scys, serum cystatin C.

**Table 4:** Diagnostic accuracy of Scys in predicting AKI at different time points.

Time	Study number	Sensitivity (95% CI)	Specificity (95% CI)	DOR (95% CI)	*I*^*2*^	AUROC (95% CI)
All settings 0 h	12	0.79 (0.70, 0.86)	0.82 (0.74, 0.88)	17 (9, 35)	92	0.88 (0.84, 0.90)
1–12 h	9	0.75 (0.70, 0.80)	0.72 (0.68, 0.76)	8 (5,12)	0	0.80 (0.76, 0.83)
24 h	16	0.82 (0.69, 0.90)	0.83 (0.76, 0.89)	23 (9, 57)	95	0.89 (0.86, 0.92)
48 h	7	0.76 (0.60, 0.88)	0.87 (0.76, 0.93)	21 (5, 58)	94	0.89 (0.86, 0.92)
1–6 h after cardiac surgery	5	0.73 (0.65, 0.80)	0.68 (0.62, 0.74)	6 (4, 9)	100	0.77 (0.73, 0.80)
12–24 h after cardiac surgery	6	0.85 (0.72, 0.92)	0.80 (0.68, 0,89)	23 (7, 77)	7	0.90 (0.87, 0.92)

Abbreviations: AKI, acute kidney injury; AUROC, the area under the receiver operating characteristic curve; DOR, diagnostic odds ratio; Scys, serum cystatin C.
